# Rapid methods for identifying barriers and solutions to improve access to community health services: a scoping review

**DOI:** 10.3399/BJGPO.2023.0047

**Published:** 2023-12-13

**Authors:** Luke N Allen, Hagar Azab, Ronald Jonga, Iris Gordon, Sarah Karanja, Nam Thaker, Jennifer Evans, Jacqueline Ramke, Andrew Bastawrous

**Affiliations:** 1 Department of Clinical Research, London School of Hygiene & Tropical Medicine, London, UK; 2 World Health Organization Regional Office for the Eastern Mediterranean, Cairo, Egypt; 3 Department of Audit and Clinical Effectiveness, Northampton Foundation trust, Northampton, UK; 4 Centre for Public Health Research, Kenya Medical Research Institute, Nairobi, Kenya

**Keywords:** health service access, rapid methods, mixed methods, review, community health services, primary health care

## Abstract

**Background:**

The advancement of universal health coverage (UHC) is largely based on identifying and addressing barriers to accessing community health services. Traditional qualitative research approaches provide excellent insights but have unfeasibly high resource requirements for most care providers.

**Aim:**

To identify, categorise, and evaluate methods that have been used to identify barriers to and/or solutions for improving access to community-based health services, grounded in engagement with affected communities, excluding approaches that take >14 days.

**Design & setting:**

This was a scoping review.

**Method:**

Following Joanna Briggs Institute (JBI) guidelines, a search was undertaken using the Cochrane Library, Ovid MEDLINE, Ovid Embase, Ovid Global Health, and Google Scholar. An information specialist designed the search, and dual independent review and data charting were used.

**Results:**

In total, 44 studies were included from 30 countries, reporting on 18 different clinical services. Thirty studies used self-described ‘rapid’ approaches; however, the majority of these did not justify what they meant by this term. Nearly half of the studies used mixed- or multi-methods and triangulation to verify early findings. All of the qualitative studies used interviews and/or focus groups, which were often supplemented with observations, document review, and mapping activities. The use of in situ snowball and convenience sampling; community members as data collectors and cultural guides; collaborative summarisation (review of findings with community members and end-users); and deductive framework analysis expedited the research processes. There were no data on costs.

**Conclusion:**

There are a wide range of methods that can be used to deliver timely information about barriers to access. The methods employed in the articles reviewed tended to use traditional data collection approaches in innovative ways.

## How this fits in

There have been abundant calls to routinely engage communities as part of extending access to health services, but most organisations have very limited time and resources to dedicate to this work. This study found that it is possible to rapidly obtain insights from those at the fringes. These assessments could play an important role in extending health service access to marginalised communities.

## Introduction

Extending universal health coverage (UHC) has been described as central to achieving the Sustainable Development Goals.^1^ As most health interactions take place in primary care, there is growing interest in understanding and tackling barriers to accessing these community-based services.^2–4^

Previous research has demonstrated the ubiquity, inequity, and impact of poor access to health care across numerous settings and service domains.^5–7^ The ascendant principles of primary health care (PHC) have focused attention on equitable access to community-based health services, grounded in community engagement and empowerment.^3,8,9^ As such, managers are facing increasing pressure to ensure that the services they run are accessible to all. Given that the factors influencing access are complex and unique in every setting,^10^ health managers and policymakers require tools to rapidly and cost-effectively identify local barriers and elicit potential solutions as a core part of routine health service provision.^11^

Seminal conceptual models of access stress both supply and demand-side factors;^10,12–14^ however, attempts to redress poor access seem to disproportionately focus on eliciting the views of those on the supply side.^15^ The World Health Organization (WHO) noted that it is invariably *‘experts who identify the problems and formulate interventions, while the problems and solutions as perceived by those at particular risk rarely constitute the base for action’*.^16^ It is increasingly recognised that efforts to improve access and attendance should be grounded in engagement with affected communities.^3,16,17^

Traditional qualitative data collection approaches, including key informant interviews (KII), in-depth interviews (IDI), ethnographic observations, and focus group discussions (FGD), commonly take many months to plan, execute, analyse, and report.^11,18,19^ High time, expertise, and resource requirements can be prohibitive for managers seeking rapid data to understand and address local issues with negligible time and resources to spend on research activity.^11,20–22^ While some forms of surveys and other quantitative approaches can be deployed relatively quickly and inexpensively, these methods are not best suited for exploring perspectives on barriers and potential solutions.^23,24^

Ideally, health service managers would be able to deploy rapid, affordable, and methodologically robust tools to engage with affected communities to elicit barriers and solutions to improve access. Such tools would have very wide application across a broad range of settings; support the development of PHC-oriented systems that are built on community engagement; and equitably extend UHC.

### Aim and objectives

This study aimed to identify, categorise, and evaluate rapid methods currently in use to identify barriers to and/or solutions for improving access to community-based health services, grounded in engagement with affected communities. For each method the study aimed to document the approach to sampling and recruitment; data collection, integration, and analysis; as well as time and resource requirements.

## Method

### Protocol and guidelines

A scoping review was chosen to be performed because this is the most appropriate method for mapping the *’extent, range, and nature of research activity in a particular field’*.^25–28^ A published protocol^29^ and the Joanna Briggs Institute methodology, based on the principles of Arksey and O’Malley and Levac *et al*, were followed.^30–32^ The Preferred Reporting Items for Systematic Reviews and Meta-Analyses (PRISMA) checklist extension for scoping reviews was used (PRISMA-ScR) to report the findings.^33^

### Eligibility

The core concept was the methods used for engaging intended service beneficiaries to elicit their perceptions of barriers to access, and/or generating ideas for service modifications that could improve access. Methods seeking to engage with those who were eligible for a given service but who had not managed to attend were focused on. Methods were excluded that sampled exclusively from attendees. Methods were included where engagement activities targeted intended beneficiaries of any non-digital community-based health service in any country, serving any need. The review was not limited to any specific population, culture, or geography.

The study focused on rapid methods, starting with an essentially arbitrary threshold, *‘methods that can be used to deliver a list of barriers and potential solutions within 14 days or less’*.^29^ It was noted that non-health sectors routinely deliver qualitative findings within a matter of weeks^34^ with timeliness, validity, and accuracy sufficient to justify $476 billion of market research spending in 2021.^35^ There is evidence that policymakers and health programme managers want — and to some extent expect — answers to health service research questions within a matter of days, so that norms and expectations around the term ‘rapid’ differ depending on context.^11,20,21,36^

Given that few definitions of rapid research use concrete time thresholds^37^ and that it is not standard practice for research articles to report the length of time taken between starting fieldwork and generating findings, studies were included that did not state how long they took, as long as they met all other inclusion criteria. Studies and approaches were divided into those that specifically used the term ‘rapid’ or a synonym to describe their approach versus studies and approaches that did not use these terms.

The focus was on access to existing community-based services. Table 1 sets out the inclusion and exclusion criteria. Systematic reviews were excluded but their reference lists were searched and any underlying primary studies that met the inclusion criteria were included. The present study included articles published in any language since 1978; the year of the Alma-Ata Declaration on Primary Health Care.^8^ While the focus was on qualitative methods, quantitative methods were not exluded.

**Table 1.** Summary of inclusion and exclusion criteria

Inclusion criteria	Exclusion criteria
Methods that elicit barriers to access and/or solutions from intended service beneficiaries or their proxies (for example, parents and carers) Established community-based services Empirical research	Methods that exclusively engage with service providers or policymakers Methods that exclusively engage with people who have managed to attend a service or health facility (service users) Methods that engage with a mix of intended service beneficiaries and service users/providers, but do not provide disaggregated findings for intended beneficiaries Methods that explicitly state that they take >14 days between starting fieldwork and generating findings Inpatient hospital services Experimental or pilot services Fully digital services Services that do not require any interaction with a clinician Enforced or compulsory services Letters, reviews, conference abstracts, non-empirical research, and methodological texts Published pre-1978

### Search strategy

The search strategy was designed by an information specialist (IG) and built around rapid community-based methods and access to health services.^26,27^ The search focused on the following: themes of access and differential access; barriers and solutions; community setting; types of research; and exclusion criteria. The Cochrane Library, Ovid MEDLINE, Ovid Embase, Ovid Global Health, and the first 20 pages of Google Scholar were searched. The search strategy, including all identified keywords and index terms, was adapted for each included database and/or information source. Box 1 presents the search strategy for MEDLINE and Supplementary Appendix S1 presents the tailored search strategies for all databases. The reference lists of included studies and relevant systematic reviews were checked to identify any additional potentially relevant reports of studies. Key authors were contacted to uncover additional or upcoming studies.

Box 1 **Search terms used for MEDLINE**

- Health Services Accessibility/
- Health Equity/
- Social Determinants of Health/
- (social adj2 determinant adj2 health$).tw.
- ((health$ or social$ or racial$ or ethnic$) adj5 (inequalit$ or inequit$ or disparit$ or equit$ or disadvantage$ or depriv$)).tw.
- (disadvant$ or marginali$ or underserved or under served or impoverish$ or minorit$ or racial$ or ethnic$).tw.
- barrier$.tw.
- (solution$ or improve$ or strateg$ or access$ or challeng$).ti.
- Community-Based Participatory Research/
- Community-Institutional Relations/
- (communit$ adj3 (engag$ or participat$)).tw.
- CBPR.tw.
- (participat$ adj2 health adj2 research).tw.
- (communit$ adj2 academic adj2 partnership$).tw.
- (collective adj2 empower$).tw.
- (equity adj2 mobili$ adj2 partnership$ adj2 communit$).tw.
- (ethnograph$ or communitarian$).tw.
- Interviews as Topic/
- Patient Health Questionnaire/
- Self Report/
- Q-Sort/
- Q-Sort.tw.
- Q-methodolog$.tw.
- (system adj2 dynamic adj2 model$).tw.
- (nominal adj2 group$ adj2 technique$).tw.
- or/1–25
- Problem Solving/
- ((rapid$ or agile) adj2 (appraisal$ or assessment$ or approach$ or evaluation$ or evaluate$ or technique$ or tool$ or method$ or research$)).tw.
- or/27–28
- 26 and 29
- in vitro.tw.
- (assay$ or microb$).tw.
- Critical Care/
- or/31–33
- 30 not 34
- limit 35 to humans
- limit 36 to (comment or editorial or letter)
- 36 not 37
- limit 38 to yr="1978 -Current"

### Evidence selection

All identified citations were collated and uploaded into Covidence (Veritas Health Innovation) and duplicates were removed. Abstracts and full texts were screened by two independent reviewers (HA and RJ) and studies that did not meet the inclusion criteria were excluded. Disagreements were resolved through consensus-based discussion and consultation with a third reviewer (LA) where necessary.

### Data charting

Two reviewers independently extracted study characteristics and data from the included studies using a form developed for this scoping review (see Supplementary Appendix S2). The form was piloted and refined during the process of extracting data from the first five articles to align it with the types of evidence that were being presented, namely the participants, concept, context, study methods, and key findings relevant to the review question (Box 2).

Box 2 **Extracted data**

- Article characteristics and study type
- Type of approach (for example, focus group) and description
- Ethics and governance requirements
- Sampling and recruitment methods
- Data collection approach
- Main output, if anything other than a prioritised list of potential service modifications
- Resource requirements:
  – Number of personnel, and essential skills or level of training
  – Number of days for each person, full-time equivalent
  – Total number of days taken from conception to findings including planning, recruitment, engagement, and analysis stages
  – Equipment
  – Total financial cost
- Framework used to structure interaction and elicit barriers and solutions
- Level of community participation
- Power relations, prevailing knowledge, and beliefs and cultural barriers, as described by the authors

All of the items identified in the original protocol were retained, but the ordering and wording of some items were reworked. The corresponding author of all articles were contacted to request missing or additional data. The lead author was also contacted if no response was received from the corresponding author within 10 days.

The level of community participation for each study was assessed using definitions set out in the WHO Europe toolkit on social participation (Box 3).^16^ These four approaches are based on those codified by the International Association for Public Participation: ‘inform’, ‘consult’, ‘involve’, ‘collaborate’, and ‘empower’, noting that inform and consult are combined by WHO under the ‘community-based’ approach.^38,39^ Each form of community engagement has legitimacy in its own right, and the most appropriate level for a given project depends on the aims and available resources.^39^ Given that the focus is on methods for identifying problems and potential solutions (that is, stopping short of implementation), the authors expected that most included studies would be community-oriented.

Box 3 **The four levels of community participation^16^**

- **Community-oriented**: the community is informed and mobilised to participate in addressing immediate short-term concerns with strong external support.
- **Community-based**: the community is consulted and involved to improve access to health services and programmes by locating interventions inside the community with some external support.
- **Community-managed**: there is collaboration with leaders of the community to enable priority settings and decisions from the people themselves with or without external support of partners.
- **Community-owned**: community assets are fully mobilised and the community is empowered to develop systems for self-governance, establish and set priorities, implement interventions, and develop sustainable mechanisms for health promotion with partners and external support groups as part of a network.

The following were also extracted: any mention of power imbalances between researchers and community-intended service beneficiaries; acknowledgements of prevailing local knowledge; and beliefs and cultural barriers to collaboration between the community members and research team. This was based on the recommendations of Turk *et al* ^17^ and a large systematic review on community participation in health systems research, which found these important issues to be chronically overlooked.^15^

### Data analysis and presentation

A narrative descriptive synthesis without meta-analysis was conducted. The synthesis was stratified by methodological approach and presented a summary table of individual study characteristics. As mentioned above, approaches were separately analysed that used ‘rapid’ or other synonyms to describe themselves. In keeping with usual practice for scoping reviews, methodological quality assessment of included studies was not conducted.^32,40^

## Results

### Study characteristics

**Figure 1.**
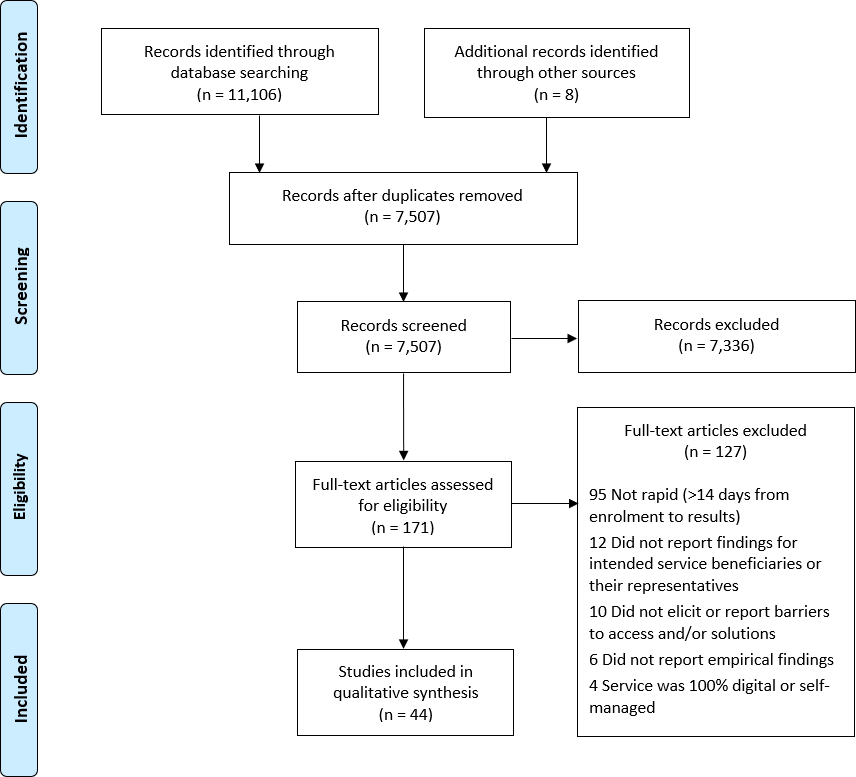
PRISMA diagram

The searches returned 7507 unique records. After excluding irrelevant articles based on title and abstracts, 171 full texts were screened with moderate agreement (Cohen’s kappa 0.47). In total, 68 authors were emailed to establish how many days their research approach took as it was not clear from the full text; 15 replies were received. All studies were included where the time taken to conduct the study was ambiguous but all other inclusion criteria were met (43 studies). A single study^41^ that stated it took a length of <14 days to complete was also included (totalling 44 studies; Figure 1).

**Figure 2.**
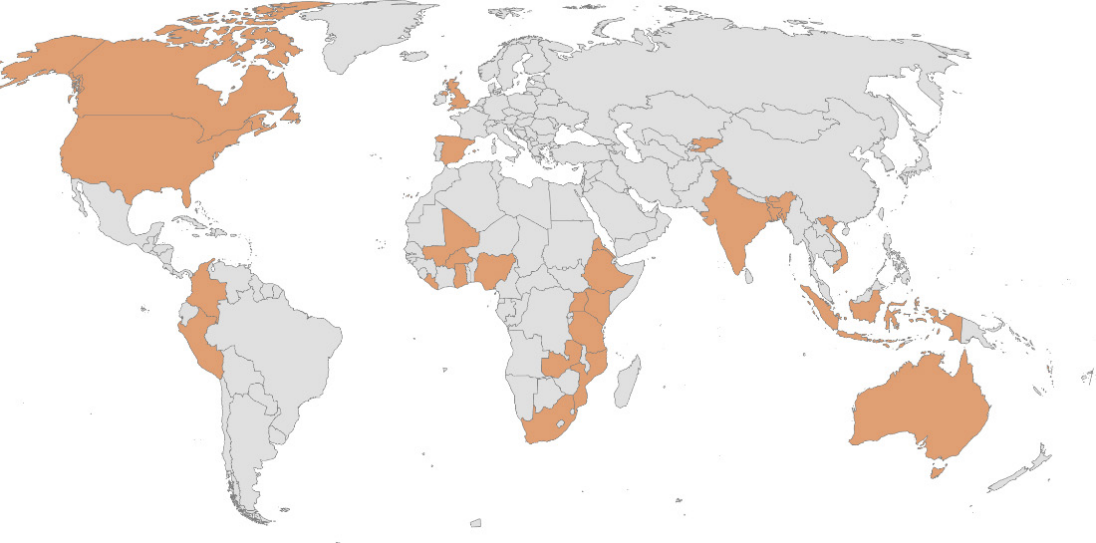
Countries represented in the scoping review

Across the 44 included studies,^41–84^ 30 countries were represented, with 19 studies (43%) based in high-income countries^41,48,52,53,55,56,63,65–67,70,71,73,74,76,79,81,82,84^ and the remaining 57% based in low- and middle-income countries (LMICs).^42–47,49–51,54,57–62,64,68,69,72,75,77,78,80,83^ Overall, 12 studies came from the US;^41,48,52,56,65–67,73,74,76,81,82^ four from India;^47,64,72,78^ two each from Australia,^55,63^ Bangladesh,^46,57^ Colombia,^53,58^ Indonesia,^50,69^ Mozambique,^45,62^ Nigeria,^42,79^ the Philippines,^44,60^ and Mali;^43,45^ and one each from Bhutan,^59^ Burkina Faso,^42^ Canada,^75^ Eritrea,^68^ Ethiopia,^49^ Georgia,^84^ Ghana,^54^ Kenya,^77^ Kyrgyzstan,^43^ Liberia,^80^ Papua New Guinea,^51^ Peru,^43^ South Africa,^83^ Spain,^71^ Tanzania,^43^ Uganda,^42^ the UK,^70^ Vanuatu,^61^ Vietnam,^44^ and Zambia (Figure 2).^45^ Four studies were conducted in multiple countries^42–45^ and the remainder focused on single countries.

**Figure 3.**
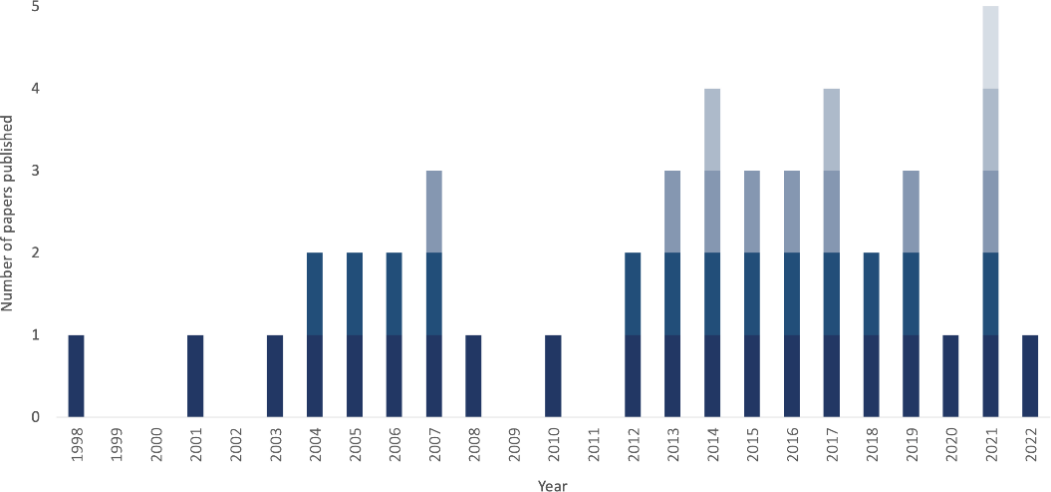
Year of publication for the included studies

Nearly three-quarters of studies (73%) had been published since 2010 (Figure 3).^41–44,46–53,56–65,67,68,71,73–78,80^ All studies were published in English.

Supplementary Table S1 summarises the study characteristics of the individual studies, dividing them into the following two groups: the 30 studies that used methods described as ‘rapid’; and the 14 studies that did not use this term. It is noted again that only one study^41^ explicitly stated that it took <14 days and that a number of the studies from the second group may well have taken >14 days to complete, but this was not able to be ascertained definitively.

### Ethical review

A large number of studies (59%) obtained ethical review from university ethics committees and, where required, national institutional review boards.^41–43,46–68^ Bedford *et al* obtained ethical approval from local *‘county health teams’*, with *‘support’* from the UNICEF Country Office (2017), and 14 studies did not provide any information on ethical review.^44,45,55,69–80^

Shimkhada *et al*’s Twitter study was exempted by the University of California, Los Angeles university ethics board.^81^ Othieno obtained written consent before conducting IDIs and FGDs with immigrants and refugees living with HIV, but stated that their organisation (the Minnesota HIV Planning Council) did not require external ethical review for this or any other needs assessments.^82^ Cook *et al* stated that ethics review was not required for their survey of barriers to cataract services because the activities *‘were planned as a component of the ongoing Vision 2020 cataract case finding in the district’*.^83^

### Services

**Figure 4.**
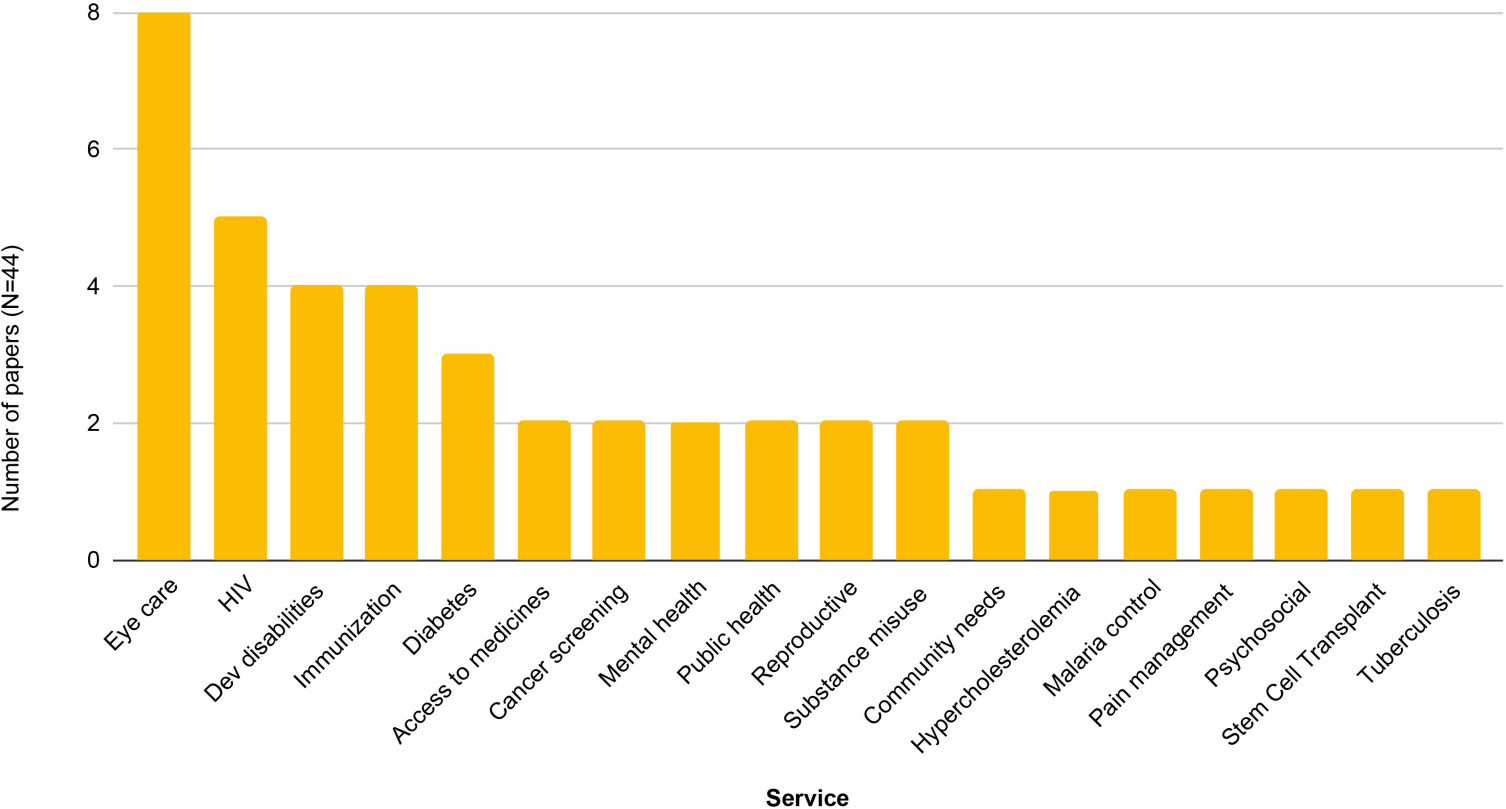
Number of studies assessing each type of service

The studies reported on 18 clinical services. The most commonly studied service was eyecare (18% of all studies); eight of these studies used the Rapid Assessment of Avoidable Blindness (RAAB) or aligned methods.^51,58,59,68,72,78,79,83^ Many more RAAB surveys were screened but excluded because they did not report barriers or stated that they took >14 days to complete. The next most commonly assessed service was HIV (11% of all included studies),^49,63,73,77,82^ followed by developmental disabilities (9%)^54–56,60^; immunisation (9%);^46,52,62,80^ diabetes (9%);^43–45,84^ access to medicines (5%);^45,47^ cancer screening (5%);^41,81^ substance misuse (5%);^71,74^ mental health (5%);^53,66^ public health intervention (5%); ^54,69^ reproduction (5%);^50,65^ and single studies assessing community needs and barriers related to access in the areas of hypercholesterolemia care, stem cell transplant, malaria, pain management, psychosocial needs, and tuberculosis (Figure 4).^42,48,57,64,67,70,76^

Eliciting barriers to access and/or solutions was the sole focus of eight of the 44 included studies (18%).^46–50,64,81,82^ The remaining 36 assessed these factors alongside other aims; for instance, Beran *et al*’s article assessed insulin availability^45^ and Brown *et al*’s article assessed community assets.^70^ All studies tended to use similar methods for eliciting barriers and solutions, irrespective of whether this was a primary or secondary aim.

### Data collection methods

Thirteen studies (30%) used surveys to assess barriers, including all of the eyecare service studies.^41,48,51,58–60,64,70–72,78,79,83^ IDIs and KIIs were the most commonly employed data collection approaches, used by all of the remaining 31 studies. Interviews were commonly combined with FGDs, cultural expert interviews, policy and administrative document review, surveys, observations, and mapping activities. Overall, 52% of the studies used a single method to elicit barriers and solutions, 41% used multiple qualitative methods, and 7% used mixed qualitative and quantitative methods (see Supplementary Table S1).

Thirty studies described their methods as ‘rapid’ (see Supplementary Table S1), and 26 of these used an established rapid-research approach (Box 4). The characteristics of these approaches are summarised in Supplementary Table S2. As has been discussed, despite using the term rapid, only one of these studies actually reported duration.^41^ The vast majority of studies used ostensibly standard approaches for recruitment, data collection, and analysis without explaining what distinguished them from ‘non-rapid’ approaches or which design features enabled the studies to be conducted faster than usual.

Box 4 **Rapid approaches mentioned in the included studies**

- PRA: Participatory Rapid Appraisal
- RA: Rapid Appraisal
- RAAB: Rapid Assessment of Avoidable Blindness
- RACSS: Rapid Assessment of Cataract Surgical Services
- RAnthroA: Rapid Anthropological Assessment
- RAP: Rapid Assessment Procedure
- RAPIA: Rapid Assessment Protocol for Insulin Access
- RARE: Rapid Assessment, Response, and Evaluation
- RAS: Rapid Assessment Survey
- RAD: Rapid Assessment of Disability
- RHA: Rapid Health Assessment
- RPA: Rapid Participatory Appraisal
- RQA: Rapid Qualitative Assessment

Two studies stated that their rapid approach traded methodological rigour for speed. Brennan and Rimba stated that their team used *‘established "quick and dirty" methods’* to gather mixed data *‘in a timely manner’* for their post-tsunami assessment,^69^ with ‘quick and dirty’ refering to the use of small (and therefore possibly non-representative) samples, trading ‘precision’ for ‘timeliness’. Beran *et al* espoused the use of ‘*pragmatic’* methods that *‘provide adequate information, without necessarily being "scientifically perfect"’*.^45^ These authors argued that pragmatism is an important principle for rapid assessments, alongside speed, cost-effectiveness, and the use of multiple data sources, which can be used to establish the validity and reliability of findings through the process of triangulation. None of the other rapid studies conceded any speed-related limitations or methodological trade-offs.

### Sampling and recruitment

Surveys tended to use multistage cluster random sampling, and this approach was largely driven by primary aims that were unrelated to eliciting barriers and solutions; for example, establishing generalisable prevalence rates. Studies that used other data collection approaches tended either not to report how they recruited participants or to recruit by approaching key informants within the local community and relevant health services to identify initial interviewees, and then used snowballing and in situ convenience sampling to recruit additional participants (see Supplementary Table S1). Six studies used additional methods to recruit participants: posters,^52^ flyers,^65^ social media,^63,65,81^ local organisations,^63,75^ clinics,^63^ and postcards.^76^ Very few studies provided information on who was responsible for recruitment (see Supplementary Table S2), and none provided information on the resources involved in terms of time.

Among the subset of self-described ‘rapid’ approaches, three studies recruited participants via adjacent services^47,52,53^ and seven studies recruited convenience or snowball samples by directly approaching people within the community of interest.^41,46,57,69,73,74,82^

All of the studies that used qualitative methods employed purposive sampling to the extent that they aimed to recruit a range of different voices from the target population of intended service beneficiaries, often focusing on those who were deemed vulnerable or marginalised.^41,46,57,69,70,73,74,82^

### Sample sizes

None of the included articles provided a justification for their sample sizes apart from Jones *et al*, who continued interviewing until achieving thematic saturation.^67^ Several of the research teams conducted >100 interviews, often supplemented with observations and surveys to identify barriers that were deliberately generalisable to the entire population of intended service beneficiaries.^43–46,62,69,80^ Elwy *et al*, Cusack *et al*, Brown *et al*, and Hill *et al* used interviews and FGDs with smaller numbers of participants but retained the same focus on population-level generalisability of findings.^52,54,70,74^ Other teams hewed to more traditional qualitative approaches, using IDIs, KIIs, and FGDs to gather rich data from small numbers of participants, trading broader transferability for thick description of the perceptions and experiences of these participants.^47,50,53,57,73,82^

### Data integration and analysis

All studies that employed Participatory Rapid Appraisal (PRA),^57^ Rapid Assessment Procedure (RAP),^50^ Rapid Participatory Appraisal (RPA),^70^ Rapid Appraisal, Rapid Assessment, Response and Evaluation (RARE),^73,74,82^ or Rapid Assessment Protocol for Insulin Access (RAPIA)^44,45^ used triangulation to check the reliability or validity of findings obtained from different approaches. Of the two different ways that triangulation is generally used in mixed-methods research,^85,86^ it seemed that most of the studies described a process of corroborating findings, rather than using different methods to gain a more complete picture of a given phenomenon, although insufficient information was provided to be certain.

Three of the four mixed-methods studies did not specify how quantitative and qualitative data were integrated.^44,45,62,73^ Cusack *et al* used a template analysis approach to integrate data around related themes within each domain.^74^

The single-method quantitative surveys both used simple descriptive statistics, while all but one of the 21 single-method qualitative studies used thematic analysis (see Supplementary Table S1).^53^ Three ‘rapid’ studies used regular research team debrief sessions, which included lay data collectors and service providers to *‘discuss and corroborate findings’*,^73^ *‘summarise key themes and observations’*,^46^ and *‘review and verify’* the research notes and emerging findings.^80^

Nicosia *et al* used an unnamed and unreferenced analytical approach *‘developed for rapid health services and implementation research’*.^76^ This involved pasting interview data into an *‘analytic matrix template’* in Microsoft Excel that organised responses by interview theme. Several other rapid approaches used similar frameworks and deductive analytical tools, which are likely to expedite the analytical process in comparison with inductive coding approaches. Cusack *at al* used *‘template analysis’* in their RARE assessment, but provided no reference or further information on what this entailed.^74^ Acosta *et al* used the RAP approach of pasting relevant quotes into a unified matrix with one row per participant, and one column per domain.^53^ Bam *et al* used a similar deductive approach to analysis, collating IDI and KII quotes with lists of barriers obtained from a mapping exercise in a single data matrix. The research team used colour-coded highlighting to apply *a priori* codes, although it is not clear how these codes were developed.^57^

Elwy *et al* analysed videocall IDI and FGD data using a *‘rapid, deductive directed content analysis approach’* described by Hsieh and Shannon,^87^ which involved populating an *a priori* coding framework (comprised of four domains and 40 subdomains), taken from an existing framework on barriers to accessing vaccination.^52^ Jones *et al* developed an *a priori* codebook based on their study’s undergirding framework, stakeholder summaries, and their interview guide domains.^67^

### Costs and resources

None of the 44 articles reported any data on costs and none of the authorship teams provided these data via email. Only one study mentioned equipment requirements (audiorecorders^55^), and only five studies stated how many people were involved in data collection: Mathias *et al* ^64^ trained 11 locals to collect data from 2400 participants; Burks *et al* ^73^ employed five data collectors and a field coordinator for their study that involved 54 participants; Patrick-Ferife *et al* used five local research assistants to collect data from 684 people,^79^ and studies led by Bedford^80^ and Watanabe^61^ both used three data collectors for studies involving 141 and 57 participants, respectively.

### Level of participation and power relations

Three studies adopted a community-based approach with research teams collaborating with locals to work as facilitators and engage with study participants.^64,67,73^ It was not possible to establish the level of participation for two of the included studies,^65,76^ and the remaining 39 used a community-oriented participation approach. Typically, this meant that the local community was informed — either electronically, by phone, or via word of mouth — of the study and invited to participate as interviewees or FGD participants. Fourteen studies engaged local community members as part of the research team. The rapid approaches used in each of these were as follows: Rapid Assessment of Disability, RARE, RAPIA, IDI and FGD, RAAB, PRA, RAP, RPA, Rapid Anthropological Assessment, and Rapid Qualitative Assessment. None of the included studies explicitly mentioned power relations or imbalances or acknowledged prevailing local knowledge or cultural barriers to participation; however, studies led by Bam, Brown, Mathias, and Burks (presented below) implicitly addressed a number of these themes through their use of rapid approaches designed to empower and partner with local community members.

Burks *et al* ^73^ employed participatory, mixed-methods action research, using four representative community members to gather data, ‘guided’ by the principle investigator. This study was based on a participatory action research paradigm. That is, there was collaboration between and within community participants at all levels of the study. A benefit of this type of research is that participants and locals of the community under study are empowered and have ownership of the study and its outcomes. The RARE methodology encourages continuous collaboration among community officials, representatives from indigenous communities, and public health workers.

Mathias *et al* ^64^ recruited data collectors who also identified with the study population. Data collectors recruited from the community of interest received a 4-day training programme that covered the interview procedures.

Studies led by Bam and Brown both used approaches with ‘participatory’ in the name. Bam *et al* ^57^ used PRA to map out perceptions of tuberculosis (TB)-related illnesses with the aid of diagrams and illustrations. Participants were also asked to identify accompanying barriers and facilitators for TB treatment. Similarly, Brown *et al* ^70^ used RPA to identify a community’s perception of its own needs and build a relationship with service providers. RPA included data collection on *‘community structure, needs, and role within existing service provision’*.^70^

## Discussion

### Summary

This scoping review identified 44 individual studies, including 30 studies that used one of 14 different self-described ‘rapid’ approaches for eliciting barriers and/or solutions to accessing community health services. Nearly half of the studies used mixed- or multi-methods, with interviews, FGD, and surveys being the most commonly employed data collection approaches, often supplemented with site visits. All of the included studies grounded their findings in the data provided by intended service beneficiaries, and a number of the rapid approaches involved local community members in data collection and analysis.

Despite many of the studies claiming to be rapid, the approaches to governance, sampling, recruitment, data collection, and analysis were orthodox for the majority of included studies. The use of team-based multi-method data collection and triangulation was used to offset truncated data collection periods, in some cases followed by same-day team-based analysis using a range of deductive tools and frameworks.

Nearly one-third of the included studies used surveys, which effectively asked participants to rank the importance of barriers that had been pre-selected by the research team. The remaining studies used qualitative methods, which are much better suited for eliciting people’s perspectives on barriers and understanding what could be done.^19,88,89^

### Strengths and limitations

This study followed international best practice guidance and a published protocol. A comprehensive search strategy was used, which was designed by an experienced information specialist, and dual independent screening and data extraction were used. However, the study has important limitations. It is very likely that a large body of experience on rapid assessments of barriers to access exists, but has not been written up and published in the peer-reviewed literature. Up to 43 out of the 44 included studies may have taken considerably longer than 2 weeks to conduct. Sixty-eight per cent of the included studies used self-described ‘rapid’ methods, but the vast majority didn’t explain or justify the use of this term. As such, this review failed to find any data on the length of time that any one approach designates for sampling, recruitment, data collection, and analysis. Critically, nor did any study (or corresponding author) provide detail on the costs and resource requirements involved.

The study deliberately focused on methods that elicit barriers and solutions by engaging with intended service beneficiaries rather than service providers or policymakers. This choice was driven by a desire to ground future assessments in community engagement, recognising that the status quo often treats service users as a ‘nice-to-have’ afterthought. In reality, the factors that influence access are multilevel and multifactorial. Findings from community-based assessments must be integrated with findings from engagement with service providers, planners, and policymakers who bring unique and important perspectives on supply-side factors, and many of the included studies did in fact engage with a wide range of stakeholders. A limitation of the review is that it stopped short of assessing how findings were used to improve service delivery and benefit service users. Future research should examine the impact of this kind of work.

A final important limitation is that the study did not set out to answer the question of whether rapid methods produce valid and trustworthy findings. There is a potential risk that the conclusions reached about barriers and potential solutions are thrown together so quickly that they oversimplify the issues, with the further risk that action on the findings leads to unintended consequences that might exacerbate inequitable access. The study found an absence of evidence that well-conducted rapid research systematically produced biased or harmful results, and the overall impression is that these tools can provide useful targeted information as an adjunct to more traditional, longer research engagements. Work by Taylor *et al* suggested that rapid approaches conducted by less-experienced researchers can deliver comparable findings to more traditional, slower methods conducted by senior qualitative researchers;^90^ however, more work is needed in this space to explore the internal and external validity of rapid methods.

### Comparison with existing literature

In the run up to 2030 health officials are coming under increasing pressure to boost access to community health services, and a core element of this work is understanding and redressing barriers. Ideally, this work would be led by highly trained qualitative researchers embedded within every community health service; however, there are nowhere near enough researchers for this work globally, nor the time or money.^91,92^ Given the scale of the need, identifying rapid and inexpensive approaches is vital.

The very concept of ‘rapid and inexpensive’ qualitative research with data collection conducted by non-specialists sounds oxymoronic to many, and is anathema to purists. However, Beebe^18^ has argued that intensive, team-based qualitative approaches that use triangulation and iterative analysis and data collection can deliver important, valid insights from ‘the insider’s perspective’ within a matter of days or weeks, rejecting the conflation of ‘rapid’ with ‘rushed’. Similarly, Johnson and Vindrola-Padrosc have argued that quick approaches don’t necessarily have to be ‘dirty’.^36^ While it does take time to build rapport, understand complexity, capture insider’s perspectives, and triangulate findings,^93–96^ rapid work can still achieve meaningful engagement, deep understanding, and decision-oriented data.^18,95^ McNall and Foster-Fishman^97^ and Trotter and Singer^98^ have argued that rapidly conducted qualitative work can even offer advantages over longer research projects in terms of promoting community engagement (by necessity) and delivering findings that can inform real-time decision making.

The 2013–2016 Ebola epidemic expedited the uptake of rapid qualitative methods and marked the first time that WHO and UNICEF recruited dedicated teams of social scientists to support their emergency responses. However, the insights provided were often difficult for policymakers to understand, and were not ultimately used to inform real-time decision making.^36^

Several of the approaches included in this review reconciled this translation issue by linking intended service beneficiaries, service providers, and policymakers through the very process of data collection and analysis. For instance Jalloh *et al* ^46^ had WHO, Centers for Disease Control and Prevention, and UNICEF partners join for team-based analysis of the transcripts from focus groups and interviews, while the studies that used the RARE approach worked closely with community members to sense-check findings and ensure that they had strong external validity to the specific community critical to the phenomena studied.^73,74,82^ Many of the studies recruited participants directly from the local community, and married IDI and FGD with observations and walks through the areas of interest.

When it comes to analysis, the deductive framework approaches used by many of the rapid models may be faster than inductive coding. However, the important work of selecting the most appropriate *a priori* framework effectively shifts some of the burden of analysis to pre-data collection rather than eliminating it completely. The real benefit may be that once the work of developing a methodologically sound coding framework is complete, people with less qualitative expertise can potentially lead elements of data collection and analysis. This could see centralised teams of qualitative researchers developing coding frameworks for all services in a given context, and the supervising of the collection and analysis of data by non-experts.

In terms of identifying an appropriate *a priori* conceptual framework, a large number have been developed for health service access.^10,12–14,99–101^ Many adopt multidimensional views of the patient and provider factors that influence whether people receive the care they need, and highlight the importance of context.^10,13,99,102–104^ Levesque *et al*’s model is one of the most commonly used, and lists five domains and related abilities that could be used to develop codes for deductive analysis.^99^ Obrist *et al* ^104^ have identified an aligned set of domains, along with five sets of livelihood assets that can be used to structure understanding of barriers to accessing health services in low-income settings.

Taylor *et al* ^11^ have previously suggested that the time a qualitative research project takes can be reduced by allowing less time between data collection episodes; for example, conducting all interviews on the same day, using multiple team members if necessary; reducing data management time by eschewing the transcription process and using notes, summaries, mind maps, and untranscribed audiorecordings instead; and speeding up the analysis phase by using one-page summaries to explore large datasets.

When surveys are used, it is important that the pre-defined options are based on empirical qualitative work that can be generalised to the population in question. There is a high risk that predefined lists offered to participants may not contain the most important barrier or solution for that context. Some surveys traverse the gap between quantitative and qualitative approaches by presenting ‘white box’ questions that allow responders to provide free-form perceptions of the barriers they face in their own words. However, without an interviewer, the opportunity to paraphrase questions, probe for more information, and observe body language is lost, limiting the value of the data.

Sampling in qualitative research does not aim to establish a representative sample for the sake of statistical inference, but rather to identify a specific group of information-rich people, which enables the theoretical generalisability of findings to other similar cases.^19^ In qualitative research, participants are purposely selected and included in the research based on their ability to extensively explore a certain topic or phenomenon. The researcher is expected to select a wide range of responders with access to extensive knowledge that can yield in-depth understanding rather than empirical generalisations.^105,106^ While many of the included studies interviewed >40 participants (and in some cases hundreds), this is unusual for qualitative research. Data and thematic saturation can be reached after interviewing 10–20 people,^107,108^ although qualitative sample size adequacy is ultimately driven by the complexity of the research question and heterogeneity of the target population.^19,105^

In George *et al*’s^15^ systematic review of 260 papers that described more than nominal community participation in health systems research, community members helped to implement interventions in 95% of the included studies, but only contributed to the identification or description of the underlying problem in 18% of the studies. The present study deliberately set out to identify approaches that specifically gather and analyse data from intended service users, and found that this work is being done across a wide range of settings and services. The vast majority of the included articles took a community-oriented approach, and three used a community-based approach, working alongside community members to collect and analyse data.

Oliver *et al* ^109^ have cautioned that coproduction brings costs as well as benefits, and these affect the research itself, the research process, and pose professional and personal risks for researchers and stakeholders, as well as *‘risks to the wider cause of scholarship’*. The take-home message is to carefully reflect on the aims and requirements for each unique project and then design the approach appropriately.

For research projects, the process of seeking and obtaining ethical review and ultimate approval for data collection is essential to protect participants and data collectors from harm. However, it can take many months and is often difficult to navigate for the uninitiated, including the average community health service manager. Even mature and well-resourced systems, such as that operated by the UK NHS Health Research Authority (HRA) are complex and take up to 60 days to deliver an initial opinion after receiving all the required documentation.^110^ Projects led by researchers affiliated with a university will often require approval from their university committee as well as the national committee.

The HRA states that formal research ethics reviews are only required for data collection that seek to extrapolate findings to a wider population. In contrast, service evaluation projects (and service improvement or development projects) do not require formal research ethical review.^110,111^ Among the included studies, 15 did not mention ethics at all^44,45,55,69–79,84^ and two studies explicitly stated that review was not required because their data collection activities were part of routine health service delivery and evaluation processes; however, they did seek written informed consent from participants.^82,83^ The take-home message is that rapid projects seeking to identify issues within a local service do not necessarily need to obtain external ethical review, although advice should be sought before proceeding.

McNall and Foster-Fishman^97^ have argued *‘the timeliness of information is no less critical than its accuracy’*, and the present review has identified a number of design features that can reduce the time taken between posing the original research question (in this case *‘what barriers prevent intended service beneficiaries from accessing the services they require and what could be done about it?’*) and delivering findings (Box 5).

Box 5 **Recommendations**

- Ask whether formal ethical review is needed. Service evaluation projects generally do not need review unless they seek to extrapolate local findings to a wider population.
- Recruit in situ, directly approaching participants rather than using passive approaches such as posters and adverts.
- Collect data at the point of recruitment, and aim to collect all data within the shortest possible amount of time.
- Use multiple forms of data collection to triangulate findings, such as direct observations, walks, site visits, interviews, and focus groups.
- Use teams of data collectors if possible, and consider working with community members who have expert local and social knowledge.
- Consider analysing data directly after collection, working from notes and audiorecordings, if appropriate.
- Analyse findings iteratively and collectively in real-time with local community members who can sense-check the findings and help identify further confirming or disconfirming cases.
- Aim to involve the ultimate users of the recommendations (that is, policymakers and service managers) in the process of analysis and the development of recommendations.
- Consider using *a priori* deductive framework approaches for data collection and analysis.
- Where appropriate, aim to compile all relevant findings on a single sheet to summarise large and complex datasets.

### Implications for research and practice

This scoping review identified a large number of research design innovations that can speed up the process of exploring barriers and potential solutions to improve access to community health services. However, the paucity of data on costs and the exact number of days that each step takes limits the ability to identify a dominant approach from the 14 different self-described ‘rapid’ methods. A number of studies were found where ‘rapid’ was a misnomer, with the term being used to describe traditional research techniques with no explanation for how or why results were obtained any faster than normal. Among the remaining studies, a common set of design features have been identified that may reduce the time taken to recruit participants and collect and analyse data (Box 5). Future research should evaluate whether approaches that utilise these strategies produce timely and robust findings, ideally with resources and cost data. Finally, a wide range of studies were found that ground the work of understanding barriers to access in the experiences and perspectives of intended service beneficiaries themselves. It is hoped that future work in this area continues to engage affected communities in the planning, execution, interpretation, and application of rapid research intended to equitably extend health for all.

## Data

The underlying studies are all cited in the reference list. All of the study tools are available in the supplementary material.
